# Mn^2+^/Yb^3+^ Codoped CsPbCl_3_ Perovskite Nanocrystals with Triple‐Wavelength Emission for Luminescent Solar Concentrators

**DOI:** 10.1002/advs.202001317

**Published:** 2020-07-27

**Authors:** Tong Cai, Junyu Wang, Wenhao Li, Katie Hills‐Kimball, Hanjun Yang, Yasutaka Nagaoka, Yucheng Yuan, Rashid Zia, Ou Chen

**Affiliations:** ^1^ Department of Chemistry Brown University 324 Brook Street Providence RI 02912 USA; ^2^ School of Engineering and Department of Physics Brown University 184 Hope Street Providence RI 02912 USA

**Keywords:** doping, luminescent solar concentrators, perovskites, quantum cutting, triple‐wavelength emission

## Abstract

Doping metal ions into lead halide perovskite nanocrystals (NCs) has attracted great attention over the past few years due to the emergence of novel properties relevant to optoelectronic applications. Here, the synthesis of Mn^2+^/Yb^3+^ codoped CsPbCl_3_ NCs through a hot‐injection technique is reported. The resulting NCs show a unique triple‐wavelength emission covering ultraviolet/blue, visible, and near‐infrared regions. By optimizing the dopant concentrations, the total photoluminescence quantum yield (PL QY) of the codoped NCs can reach ≈125.3% due to quantum cutting effects. Mechanism studies reveal the efficient energy transfer processes from host NCs to Mn^2+^ and Yb^3+^ dopant ions, as well as a possible inter‐dopant energy transfer from Mn^2+^ to Yb^3+^ ion centers. Owing to the high PL QYs and minimal reabsorption loss, the codoped perovskite NCs are demonstrated to be used as efficient emitters in luminescent solar concentrators, with greatly enhanced external optical efficiency compared to that of using solely Mn^2+^ doped CsPbCl_3_ NCs. This study presents a new model system for enriching doping chemistry studies and future applications of perovskite NCs.

## Graphical abstract

Mn^2+^/Yb^3+^ codoped CsPbCl_3_ perovskite nanocrystals are synthesized with a unique triple‐wavelength emission covering the ultraviolet/blue, visible, and near‐infrared spectral regions. Due to high photoluminescence quantum yields and minimal reabsorption losses, the codoped nanocrystals can be used as efficient emitters in large‐area luminescent solar concentrators with superior light‐concentrating performance.

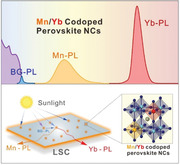

Incorporation of impurity ions (also known as the doping process) into perovskite nanocrystals (NCs) has proven as a unique and effective means to alter and enhance the magnetic, magneto‐optical and optoelectronic properties of the host perovskite NCs.^[^ ^1^, ^2^, ^3^, ^4^, ^5^, ^6^ ^]^ To date, various excellent dopant examples have been demonstrated including main group elements (e.g., Al, Ca, Sr, Bi),^[^ ^7^, ^8^, ^9^, ^10^ ^]^ transition metals (e.g., Mn, Ni, Cd),^[^ ^11^, ^12^, ^13^, ^14^, ^15^, ^16^, ^17^, ^18^, ^19^, ^20^, ^21^, ^22^, ^23^, ^24^, ^25^ ^]^ and rare earth elements (e.g., Yb, Ce, Pr).^[^ ^26^, ^27^, ^28^, ^29^, ^30^, ^31^, ^32^, ^33^, ^34^, ^35^, ^36^, ^37^ ^]^ Among all the dopant‐induced properties, the emergence of new emission bands due to the introduction of additional radiative relaxation channels of excitons has been intriguing and promising in particular.^[^ ^2^ ^]^ To this extent, doping Mn^2+^ ions into host semiconductor crystals has historically been one of the most‐studied model systems due to the possible introduction of a dopant emission channel through the d–d transition of Mn^2+^ ion centers.^[^ ^11^ ^]^ To date, Mn^2+^ ions have been successfully doped in a variety of host systems ranging from traditional bulk scale semiconductors (forming diluted magnetic semiconductors),^[^ ^38^ ^]^ conventional semiconductor quantum dots (e.g., CdSe‐, ZnS‐, ZnSe‐based quantum dots),^[^ ^39^, ^40^, ^41^, ^42^ ^]^ more recently to halide perovskite NCs.^[^ ^11^, ^12^, ^13^, ^14^, ^15^, ^16^ ^]^ Both the magnetic and optical properties of the host materials can be dramatically altered owing to the presence of few Mn^2+^ ions in the crystal lattices.

In recent years, the rare earth Yb^3+^ ion has become another “hot” doping candidate not only for fundamental studies of doping chemistry and photo‐physics, but also with high application potentials.^[^ ^27^, ^28^, ^29^, ^30^, ^31^, ^32^ ^]^ The high popularity of doping Yb^3+^ ions is largely due to the unique exciton energy relaxation process through a so‐called quantum cutting mechanism that occurs at the Yb^3+^ ions when doped in wide‐bandgap (BG) semiconductor NCs.^[^ ^27^, ^28^ ^]^ When BG energy is sufficient, a single high‐energy (typically, ultraviolet (UV) or blue) photon that is absorbed at the band edge can be split into two near‐infrared (NIR) photons at the Yb^3+^ ion centers, resulting in the emission through an f–f transition from the Yb^3+ 2^F_5/2_ excited state to the ^2^F_7/2_ ground state.^[^ ^27^, ^28^ ^]^ As a consequence, this quantum cutting process enables the photoluminescence quantum yield (PL QY) of the NCs to exceed unity, overcoming the fundamental limitation for single exciton relaxation processes.^[^ ^27^ ^]^

Current studies focus mostly on doping a single‐type of metal ions into the host perovskite NCs, while few research efforts have been made on simultaneously doping more than one type of metal ions into the same perovskite hosts and intra‐particle energy transfer processes.^[^ ^29^, ^30^, ^43^, ^44^ ^]^ In particular, despite the high popularities of each dopant on their own, no studies have been reported so far about codoping both Mn^2+^ and Yb^3+^ ions concomitantly into one perovskite NC sample. Beneficially, compared to solely Mn^2+^ doped CsPbCl_3_ NCs, the co‐introduction of Yb^3+^ dopants may lead to an increased total PL QY while maintaining the large Stokes shift for both Mn‐ and Yb‐emission bands with decreased reabsorption loss. Moreover, different natures of emission mechanisms can be involved in a single batch of Mn^2+^/Yb^3+^ codoped perovskite NCs: i) electronic inter‐band transition (BG‐PL); ii) electronic transition of ion centers within local molecular complexes (Mn‐PL); and iii) defect‐induced energy transfer and subsequent quantum cutting scheme (Yb‐PL). Therefore, codoping both Mn^2+^ and Yb^3+^ ions into perovskite NCs can serve as a unique model system for studies of host‐to‐dopant and inter‐dopant energy transfer mechanisms, as well as associated radiative and nonradiative decay pathways at each step. Most importantly, such codoped NCs with multiple emission channels hold a high potential to be applied in a wide range of applications,^[^ ^45^, ^46^, ^47^, ^48^, ^49^, ^50^, ^51^, ^52^, ^53^, ^54^, ^55^, ^56^, ^57^, ^58^, ^59^, ^60^, ^61^ ^]^ including multiplexed biological labelling and sensing,^[^ ^59^ ^]^ multi‐channel photodetectors,^[^ ^60^ ^]^ stimuli‐responsive inks for coding, encryption and decryption,^[^ ^61^ ^]^ and photon management devices.^[^ ^45^, ^46^, ^47^, ^48^, ^49^, ^50^, ^51^, ^52^, ^53^, ^54^, ^55^, ^56^, ^57^, ^58^ ^]^

Herein, we report a facile synthesis of Mn^2+^/Yb^3+^ codoped CsPbCl_3_ perovskite NCs (**Scheme** **1**) through a hot‐injection method. Owing to the completely separated energy relaxation channels among the intrinsic BG, Mn^2+^ and Yb^3+^ dopant emissions, the obtained Mn^2+^/Yb^3+^ codoped CsPbCl_3_ perovskite NCs exhibit a unique triple‐wavelength emission profile simultaneously covering UV/blue (BG‐PL), visible (Mn‐PL) and NIR (Yb‐PL) spectral regions. We show that the concentrations of each dopant can be independently controlled, resulting in facile tunability of the PL emission profiles. Both steady state and time‐resolved photoluminescence (TR‐PL) spectroscopic studies reveal the efficient energy transfer pathways from host NCs to Mn^2+^ and Yb^3+^ dopant ions, as well as a possible inter‐dopant energy transfer from Mn^2+^ to Yb^3+^ ion centers. In addition, due to the large Stokes shift with minimal reabsorption loss, and greatly increased PL QYs as compared to the solely Mn^2+^ doped CsPbCl_3_ NCs, the Mn^2+^/Yb^3+^ codoped NCs exhibit enhanced performance when applied as emitters for luminescent solar concentrator (LSC) applications. Our study presented here not only demonstrates a unique model platform for studying doping chemistry and energy transfer mechanisms for multi‐dopants systems, but also paves the way for future creations of next generation emitting materials with multiple emission channels, which hold the promise for a variety of desired applications, including lighting, sensing and solar energy harvesting.

**Scheme 1:**
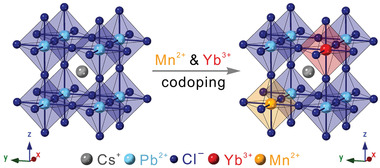
Schematic illustration of Mn^2+^/Yb^3+^ codoped CsPbCl_3_ perovskite NCs.

Mn^2+^/Yb^3+^ codoped CsPbCl_3_ perovskite NCs were synthesized following a modified hot‐injection method, where metal acetate salts and chlorotrimethylsilane (TMS‐Cl) played the roles as metal and halide sources, respectively.^[^ ^27^, ^62^, ^63^ ^]^ In a typical synthesis, CH_3_COOCs, Pb(CH_3_COO)_2_·3H_2_O (Pb‐acetate), Mn(CH_3_COO)_2_·4H_2_O (Mn‐acetate), and Yb(CH_3_COO)_3_·4H_2_O (Yb‐acetate) with various molar ratios were added to a mixture of oleic acid, oleylamine, and 1‐octadecene. The resulting solution was first heated to 120 °C under vacuum to remove water and oxygen. Subsequently, TMS‐Cl was swiftly injected into the flask at 200 °C and the reaction was kept at 200 °C for 10 s before termination by ice‐bath cooling (see the Supporting Information for details). This approach allows for a facile tunability in Mn^2+^/Yb^3+^ codoping levels by altering the stoichiometry of the Pb^2+^, Mn^2+^ and Yb^3+^ precursors fed into the reaction.^[^ ^27^ ^]^

**Figure** **1** shows the optical, morphology and crystal structure characterization results of the undoped and Mn^2+^/Yb^3+^ codoped CsPbCl_3_ perovskite NCs. The absorption spectra of both samples showed similar absorption onset and peaks, indicating no significant changes on the electronic structure of the host CsPbCl_3_ NCs after doping Mn^2+^ and Yb^3+^ ions (Figure 1A,B). As compared to the typical PL spectrum of the undoped CsPbCl_3_ NCs with a single BG excitonic emission peak at 404 nm (Figure 1A), two additional PL peaks centered at 618 and 985 nm emerged for the Mn^2+^/Yb^3+^ codoped sample (Figure 1B). These two peaks can be assigned to the ^4^T_1g_–^6^A_1g_ d–d transition of the Mn^2+^ ions and the ^2^F_5/2_–^2^F_7/2_ f–f transition of the Yb^3+^ ions, respectively.^[^ ^11^, ^27^ ^]^ Besides, a pseudo‐color map of the excitation‐dependent PL spectra of the codoped sample further revealed the triple emission bands under different excitation light (Figure S1, Supporting Information). In addition, the BG PL peak of the codoped sample showed a 30.7 meV blueshift (from 404 to 400 nm) as compared to the undoped perovskite NCs. When monitoring the emission at 618 and 985 nm, the PL excitation spectra matched well with the absorption profile (Figure S2, Supporting Information), indicating that the emergence of the two emission peaks was caused by energy transfer processes from the host CsPbCl_3_ NCs to the Mn^2+^ and Yb^3+^ dopant ions (detailed energy transfer mechanism will be discussed below). Transmission electron microscopy (TEM) measurements showed that the Mn^2+^/Yb^3+^ codoped CsPbCl_3_ NCs exhibited a uniform cubic shape with an average edge‐length of 7.2 ± 0.7 nm, slightly smaller than that of the undoped CsPbCl_3_ NCs (average edge‐length of 8.3 ± 0.8 nm) (Figure 1C,D and Figure S3, Supporting Information). The powder X‐ray diffraction (XRD) patterns of both undoped and codoped samples showed an unambiguous cubic perovskite crystal phase (space group: $\mathrm{Pm}\bar{3}m$, Figure 1E and Figures S4–S5, Supporting Information), indicating an undisturbed crystal structure of host NCs.^[^ ^11^, ^27^, ^64^, ^65^ ^]^ However, all the Bragg diffraction peaks of the codoped CsPbCl_3_ NCs shifted to larger diffraction angles, revealing a smaller lattice parameter as compared to the undoped counterpart (5.609 Å vs 5.551 Å, Tables S1–S2, Supporting Information). The XRD results indicated the proposed crystal lattice contraction after substitutions of Pb^2+^ ions (132 pm) with smaller Mn^2+^ (81 pm) and Yb^3+^ (100 pm) ions,^[^ ^66^ ^]^ in line with the optical and morphological measurements shown in Figure 1A–D.^[^ ^11^, ^28^, ^67^, ^68^ ^]^

**Figure 1:**
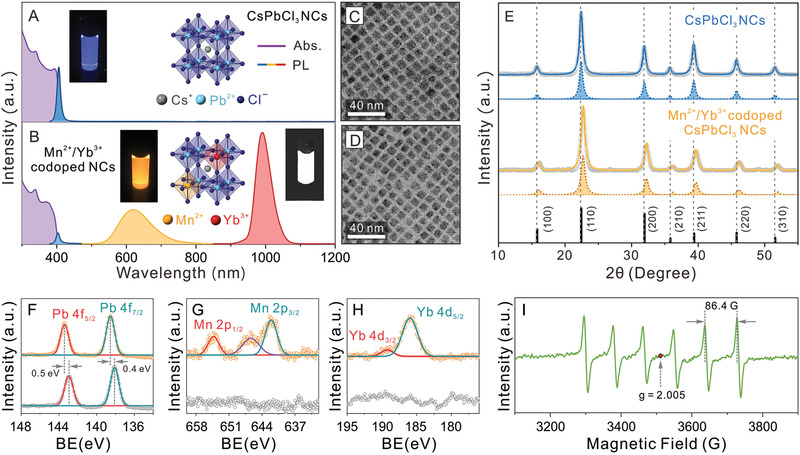
A,B) Absorption (purple), PL (blue, orange, and red) spectra of the undoped and Mn^2+^/Yb^3+^ codoped CsPbCl_3_ perovskite NCs. Insets: schematics of the perovskite crystal structures and photographs of the samples under UV illumination (365 nm) taken by a visible‐camera and a NIR‐camera with an 800 nm long‐path filter. C,D) TEM images of undoped and Mn^2+^/Yb^3+^ codoped CsPbCl_3_ NCs. E) XRD patterns of undoped (top) and Mn^2+^/Yb^3+^ codoped (bottom) CsPbCl_3_ NCs (gray), fitted curves (solid lines), and constituent peaks (dotted lines with shadows). Black bars indicate the standard peak positions of bulk CsPbCl_3_ perovskite. F–H) XPS spectra of both undoped (bottom) and codoped samples (top) for F) Pb 4f, G) Mn 2p, and H) Yb 4d peaks. BE: Binding Energy. The spectra are calibrated using C 1s peak. I) EPR spectrum for the Mn^2+^/Yb^3+^ codoped perovskite NCs.

The successful doping of Mn^2+^ and Yb^3+^ ions was further confirmed by the X‐ray photoelectron spectroscopy (XPS) and electron paramagnetic resonance (EPR) measurements. The XPS spectra of the codoped NCs showed that all the Cs 3d, Pb 4f, Cl 2p peaks shifted to higher energy side after the incorporation of Mn^2+^ and Yb^3+^ dopants (Figure 1F and Figure S6, Supporting Information), indicating the stronger Pb‐Cl interactions in the codoped NCs, consistent with the previous reports.^[^ ^7^, ^23^ ^]^ This was further evidenced by the emergence of Mn 2p and Yb 4d peaks in the codoped sample, indicating the co‐existence of Mn^2+^ and Yb^3+^ ions (Figure 1G,H).^[^ ^30^, ^43^ ^]^ While the undoped CsPbCl_3_ NCs showed EPR silence (Figure S7, Supporting Information), the Mn^2+^/Yb^3+^ codoped CsPbCl_3_ NCs displayed a set of six nuclear‐electron hyperfine splitting peaks with an average splitting constant of 86.4 G and a characteristic *g*‐factor value of 2.005 (Figure 1I), demonstrating the successful insertion of Mn^2+^ ions into the octahedra coordination environment in the cubic perovskite lattice.^[^ ^21^, ^22^ ^]^

To study the doping concentration effects on the structural and optical properties of the Mn^2+^/Yb^3+^ codoped CsPbCl_3_ perovskite NCs, we synthesized a series of samples (denoted as sample 1‐6) with varied Mn^2+^ and Yb^3+^ doping concentrations by altering the metal precursor feeding amounts (**Figure** **2**, see the Supporting Information for synthetic details). The doping concentrations in the final NCs were measured by inductively coupled plasma‐atomic emission spectroscopy (ICP‐AES) and determined to be 2.24%, 2.17%, 1.45%, 1.30%, and 1.14% for Mn^2+^ dopants ([Mn]/([Pb]+[Mn]+[Yb])), and 0.00%, 3.94%, 6.60%, 10.76%, and 15.23% for Yb^3+^ dopants ([Yb]/([Pb]+[Mn]+[Yb])) in the samples 2‐6, respectively (Figure 2I and Table S3, Supporting Information). When increasing the feeding amount, thus the doping concentration of Yb^3+^ ions in the final NCs, a decrease of Mn^2+^ doping concentration was observed despite the unchanged Mn^2+^ precursor feeding amount (Figure 2I). This result indicated a competing doping process between the two types of dopants (i.e., Mn^2+^ and Yb^3+^) during the NC formation. TEM measurements showed that all the samples possessed a uniform cubic shape with average edge‐lengths of 8.3 ± 0.8, 8.0 ± 0.7, 7.8 ± 0.7, 7.5 ± 0.7, 7.2 ± 0.7, and 6.8 ± 0.7 nm for samples 1–6, respectively (Figure 2A–F and Figure S8, Supporting Information). This gradual decrease in size was partially attributed to the increased lattice contraction effects upon increasing doping concentrations, which was further evidenced by the XRD measurements (Figure 2G,H). The XRD patterns revealed a continuous crystal lattice shrinkage with the calculated lattice constants decreased from 5.609 to 5.547 Å (Figure 2J, Figures S4–S5, S9–S12 (Supporting Information) and **Table** **1** and Tables S1–S2, S4–S7, Supporting Information) while preserving the cubic perovskite crystal phase (space group: $\mathrm{Pm}\bar{3}m$) for all the six samples.

**Figure 2:**
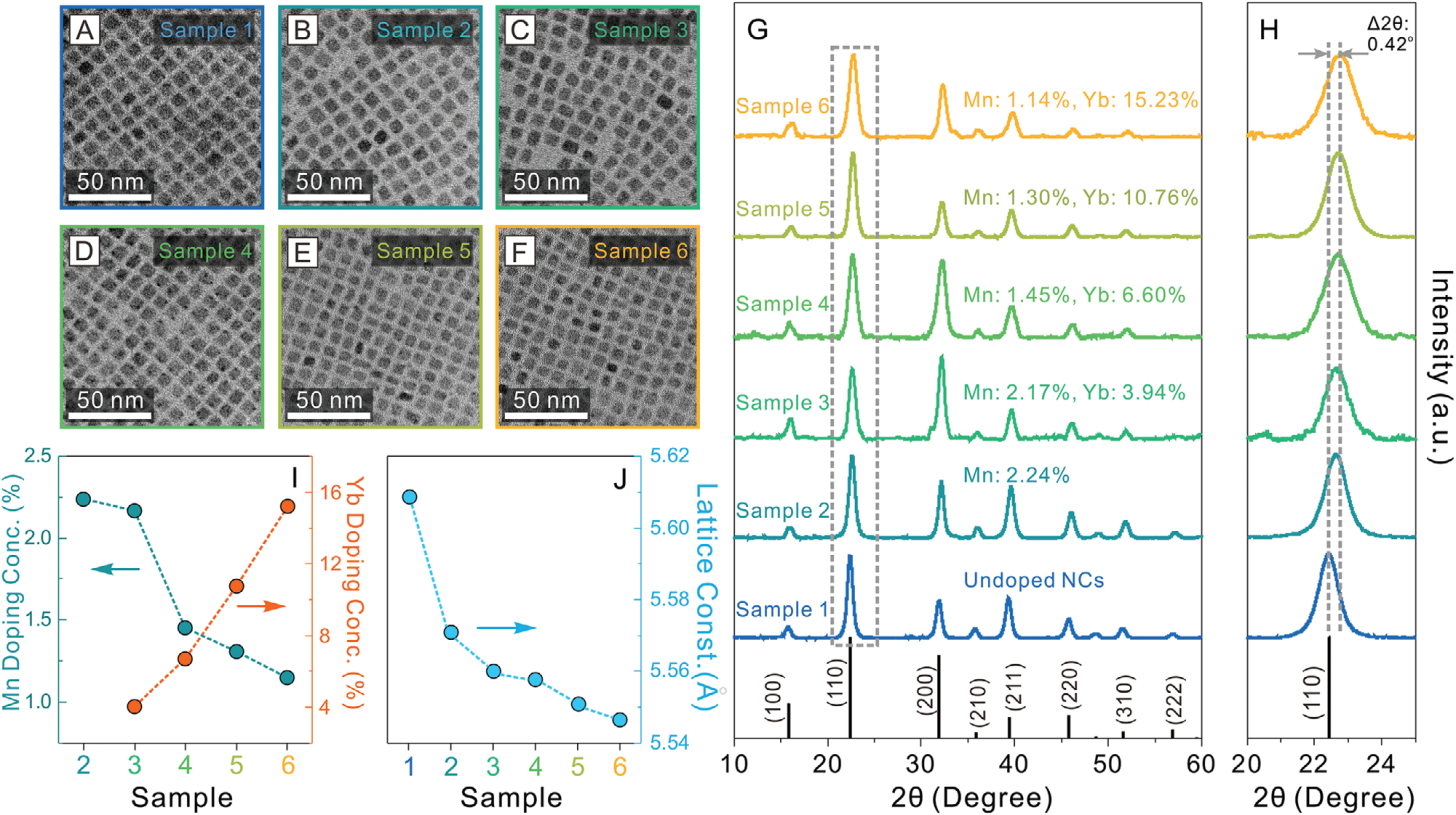
A–F) TEM images of undoped and Mn^2+^/Yb^3+^ codoped CsPbCl_3_ NCs with different doping concentrations. G) XRD patterns for different doped perovskite NCs. H) Zoomed‐in XRD patterns of the (110) diffraction peak. I) Mn^2+^‐ and Yb^3+^‐ doping concentrations of Mn^2+^/Yb^3+^ codoped CsPbCl_3_ NCs determined by ICP‐AES. J) The evolution of lattice constant of Mn^2+^/Yb^3+^ codoped CsPbCl_3_ NCs with different doping concentrations.

**Table 1:** Summary of doping concentrations (conc.), lattice constant, average PL lifetime ($\bar{\tau }$), and PL QYs for the undoped and Mn^2+^/Yb^3+^ codoped CsPbCl_3_ perovskite NCs

Sample #	Mn doping Conc.	Yb doping Conc.	Lattice constant [Å]	BG‐PL $\bar{\tau }$ [ns]	Mn‐PL $\bar{\tau }$ [ms]	Yb‐PL $\bar{\tau }$ [ms]	BG‐PL QY [%]	Mn‐PL QY [%]	Yb‐PL QY [%]	Total PL QY [%]
1	/	/	5.609	4.99	/	/	8.7	/	/	8.7
2	2.24%	/	5.571	3.26	1.33	/	3.3	45.1	/	48.4
3	2.17%	3.94%	5.560	2.34	1.22	0.97	1.2	35.3	32.5	69.0
4	1.45%	6.60%	5.558	2.05	1.11	1.11	0.9	29.7	64.6	95.1
5	1.30%	10.76%	5.551	1.86	1.02	1.14	0.5	21.5	103.3	125.3
6	1.14%	15.23%	5.547	1.36	0.96	1.42	0.4	16.3	66.3	83.0

We further characterized the optical properties of these six samples, and the results are shown in **Figure** **3**. UV–vis absorption measurements showed that all six samples displayed similar absorption spectral profiles (Figure 3A). The PL and PL QY measurements revealed that, other than sample 1 (undoped sample) and sample 2 (solely Mn^2+^ doped sample), samples 3‐6 all showed triple‐wavelength PL profiles with different PL QYs (Figure 3B,C). With increased total doping concentrations (for both Mn^2+^ and Yb^3+^ dopants, ([Mn]+[Yb])/([Pb]+[Mn]+[Yb])), the BG‐PL QY showed a monotone decrease from 8.7% to 0.4%, indicating increased energy transfer from the conduction band of the host perovskite NCs to the dopants (i.e., Mn^2+^ and Yb^3+^ ion centers) (Figure 3C and Table 1).^[^ ^11^, ^27^ ^]^ The same QY decreasing trend (from 45.1% to 16.3%) was observed for the Mn‐PL while increasing the Yb^3+^ doping concentration (Figure 3C and Table 1). Meanwhile, the Yb‐PL QY increased from 0% to 103.3% as the Yb^3+^‐doping concentration increased from 0% to 10.76% (Figure 3C), supporting a quantum cutting process at the Yb^3+^ ion centers.^[^ ^27^, ^28^ ^]^ Upon further increase of the Yb^3+^‐doping concentration to 15.23%, the Yb‐PL QY decreased to 66.3%. This QY decrease was likely caused by the increased crystalline defects and an inter‐dopant coupling induced self‐quenching effect due to the introduction of excess Yb^3+^‐dopants.^[^ ^27^ ^]^ The total PL QYs of the samples were summarized in Figure 3C (green dots). It is worth noting that the highest total PL QY of codoped sample was 125.3%, which was ≈14 times and ≈2.6 higher than those of the undoped CsPbCl_3_ NCs and the solely Mn^2+^ doped CsPbCl_3_ NCs, respectively (Table 1), and comparable to solely Yb^3+^ doped CsPbCl_3_ NCs with a similar Yb^3+^‐doping concentration (Table S8, Supporting Information).^[^ ^27^, ^28^, ^29^, ^46^, ^69^ ^]^

**Figure 3:**
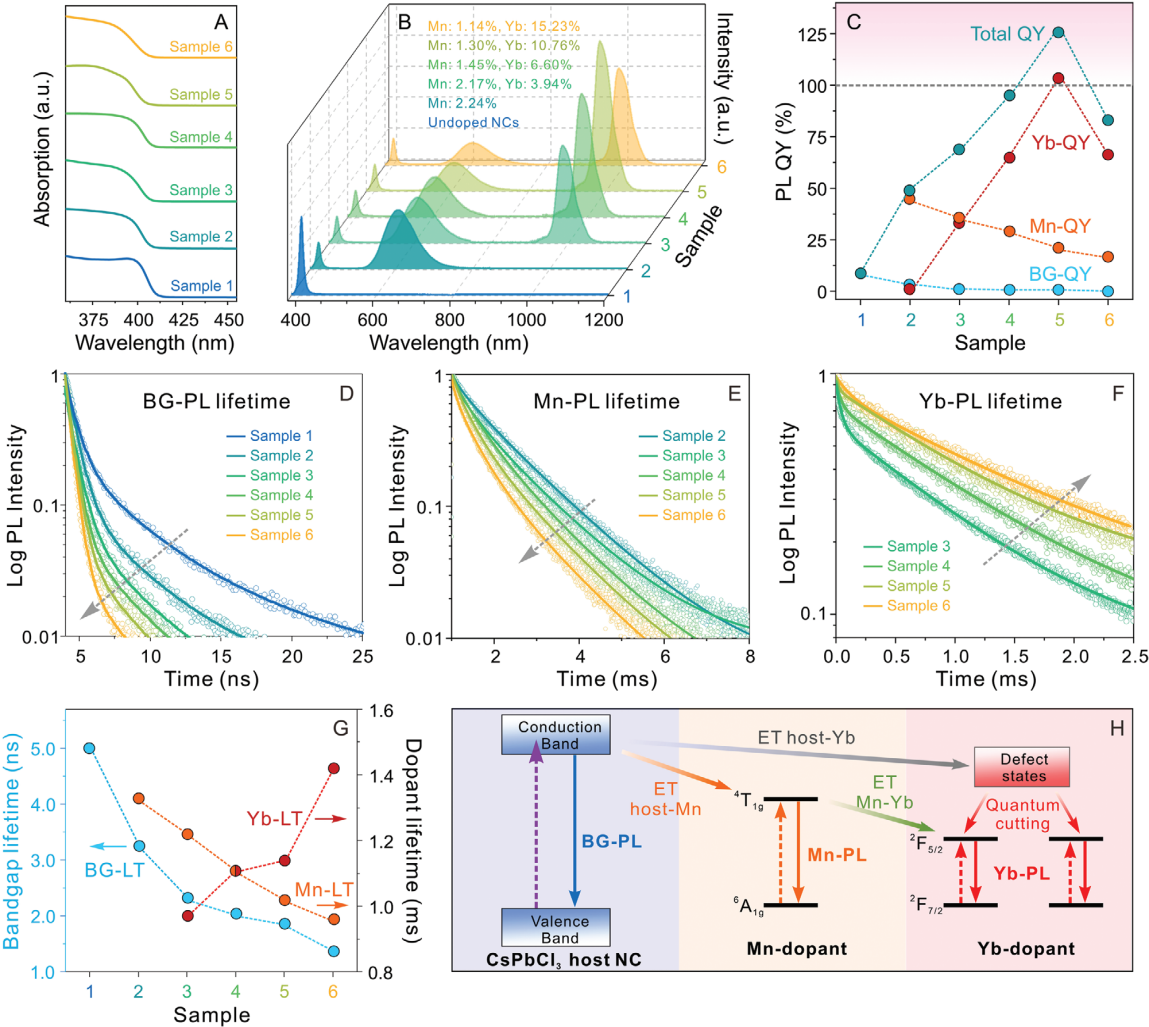
A) Absorption spectra B) PL spectra and C) PL QYs of the undoped and Mn^2+^/Yb^3+^ codoped CsPbCl_3_ NCs with different doping concentrations. D–F) Lifetime (LT) decay curves for the samples when monitoring BG‐PL (D), E)Mn‐PL (D), and Yb‐PL (F). G) The summary of the average LTs for BG (blue), Mn (orange), and Yb (red) PLs. H) Schematic of the proposed energy transfer (ET) processes in the Mn^2+^/Yb^3+^ codoped CsPbCl_3_ perovskite NCs.

TR‐PL spectroscopy measurements were carried out for all the six samples and the results are shown in Figure 3D–G and Table 1. Upon increasing the total doping concentrations, the excitonic BG‐PL decay became faster as the fitted average PL lifetime (LT) decreased from 4.99 to 1.36 ns (Figure 3D,G and Table S9, Supporting Information). This shortened PL LT was attributed to the accelerated depletion of the excitonic transitions due to the energy transfer from the host NCs to dopants (i.e., Mn^2+^ and Yb^3+^ ions),^[^ ^22^, ^27^ ^]^ in line with the PLE measurements (Figure S2, Supporting Information). Interestingly, upon decreasing the Mn^2+^‐doping concentration from 2.24% to 1.15%, the Mn‐PL LT decreased from 1.33 to 0.96 ms (Figure 3E,G and Table S10, Supporting Information), demonstrating an opposite trend reported for the LT change of solely Mn^2+^ doped CsPbCl_3_ NCs with decreased concentrations.^[^ ^15^, ^22^ ^]^ This opposite trend can only be explained by the concomitant doping of Yb^3+^ ions. The Mn‐PL LT decrease (Figure 3E,G and Table S10, Supporting Information) indicated the occurrence of inter‐dopant energy transfer process from Mn^2+^ to Yb^3+^ ions.^[^ ^29^, ^30^, ^43^ ^]^ To further prove our hypothesis, control experiments were conducted by measuring the Mn‐PL LT of a mixture solution of solely Mn^2+^ doped NCs with solely Yb^3+^ doped NCs. Negligible changes of Mn‐PL LT were observed irrespective of different mixed amounts of Yb^3+^ doped NCs (Figure S13 and Table S11, Supporting Information). Furthermore, the positive correlation between the Yb^3+^ doping concentration and the corresponding Yb‐PL LT further confirmed the enlarged population of the ^2^F_7/2_ state of Yb^3+^ ions, which is assisted by the codoped Mn^2+^ ions within the same CsPbCl_3_ perovskite host NCs (Figure 3F,G and Table S12, Supporting Information).^[^ ^29^, ^30^ ^]^

A complete picture of the photoexcitation, energy transfer and radiative pathways for the Mn^2+^/Yb^3+^ codoped CsPbCl_3_ perovskite NCs were delineated in Figure 3H. After the UV light excitation, excitons (an electron and hole pair) can be firstly photogenerated in the CsPbCl_3_ host NCs. Partial exciton recombination at the band edge gives rise to the BG‐PL. After doping with Mn^2+^ ions, a portion of the excited electrons can transfer energy to the excited state (^4^T_1g_) of the Mn^2+^ dopants through a resonant Dexter‐type energy transfer process.^[^ ^15^ ^]^ While for the Yb^3+^ dopants, the internal lattice defects would participate in the photo‐relaxation processes due to the aliovalent charge of the Yb^3+^ ions in the CsPbCl_3_ NC hosts.^[^ ^27^ ^]^ Specifically, energy transfer firstly occurs from excitons of the host NCs to the localized defects then to the neighboring Yb^3+^ ions, followed by a quantum‐cutting process.^[^ ^27^, ^28^, ^29^ ^]^ In addition, a direct energy transfer from ^4^T_1g_ electronic state of the Mn^2+^ ions to the ^2^F_5/2_ state of Yb^3+^ ions within a perovskite NC may also occur, further populating (depopulating) the excitation state of the Yb^3+^ (Mn^2+^) ions and consequently increasing (decreasing) the Yb‐ (Mn‐) PL QY as we observed experimentally (Figure 3C and Table 1). Although detailed energy transfer mechanism between Mn^2+^ and Yb^3+^ dopant centers is still under investigation, no quantum‐cutting effect should be involved in this inter‐dopant energy transfer (from Mn^2+^ to Yb^3+^) and relaxation processes due to the insufficient energy input from Mn^2+^ dopants.

Semiconductor NCs doped with Mn^2+^ ions have proven as superior emitters for LSCs (a photon managing device that can harvest, direct and concentrate light) largely owing to their reabsorption‐free property imparted by a large Stokes shift.^[^ ^45^ ^]^ In this regard, we expect that the Mn^2+^/Yb^3+^ codoped NCs exhibit enhanced solar light concentrating performance as compared to the solely Mn^2+^ doped NCs, due to the addition of Yb emission with increased PL QY.^[^ ^33^, ^46^, ^69^ ^]^ To evaluate the performance of the Mn^2+^/Yb^3+^ codoped CsPbCl_3_ NCs, we fabricated a large LSC device (dimensions: 20 cm × 20 cm × 0.5 cm) by embedding Mn^2+^/Yb^3+^ codoped CsPbCl_3_ NCs with a total PL QY of 125.3% (sample 5) in a polydimethylsiloxane (PDMS) polymer matrix (NC concentration of 0.3 wt%) (Figure 4A,B and see the Supporting Information for fabrication details). The Mn‐ and Yb‐PL spectral profiles of the NC solution sample were nearly identical to those of the NC‐embedded LSC device (denoted as Mn/Yb‐LSC) (Figure S14, Supporting Information). This indicated the homogeneous solid‐solution nature of the device with no photon reabsorption of Mn and Yb emissions and negligible scattering effect (Figure S14, Supporting Information).^[^ ^45^, ^69^ ^]^ In addition, due to the only UV light absorption (Figure 4A), the Mn/Yb‐LSC device showed a high visible‐light transmittance of 74.2% (Figure 4C and Figure S15, Supporting Information), rendering the device suitable for converting building or automobile windows into LSC‐based power generation units.^[^ ^47^, ^70^ ^]^

**Figure 4:**
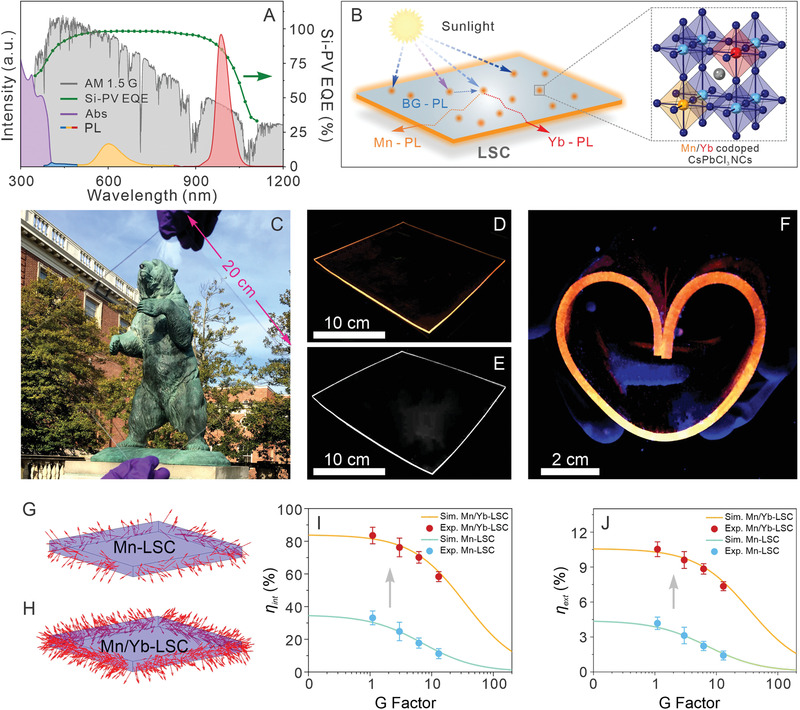
A) Absorption (purple) and PL spectra of Mn/Yb‐LSC (BG‐PL: blue, Mn‐PL: orange, and Yb‐PL: red). Green line is a plot of the external quantum efficiency (EQE) for a polycrystalline silicon (c‐Si) mini cell. The gray area is the solar spectrum. B) Schematic of the LSC model made of a polymer matrix containing the Mn^2+^/Yb^3+^ codoped CsPbCl_3_ NCs. (Inset: the crystal structure of codoped NC atomic model). C) Photograph of an LSC comprising Mn^2+^/Yb^3+^ codoped CsPbCl_3_ NCs (dimensions: 20 cm × 20 cm × 0.5 cm, NC concentration: 0.3 wt%). D,E) Photographs of the LSC device under UV illumination (365 nm) taken by a visible camera (D) and a NIR‐camera with an 800 nm long‐path filter (E). F) Photograph of the bent LSC device under UV illumination taken by a visible camera. Monte Carlo ray‐tracing simulations for LSC devices incorporating solely Mn^2+^ doped CsPbCl_3_ NCs G) and Mn^2+^/Yb^3+^ codoped CsPbCl_3_ NCs H). I,J) internal optical efficiency (*η*_int_) I) and external optical efficiency (*η*_ext_) J) of the Mn‐LSC and Mn/Yb‐LSC devices under sunlight illumination. The statue in the photograph showing the LSC in (C) appears with permission from Brown University.

The photographs of the device under UV illumination (365 nm) showed a strong light concentrating effect (to the edges of the device) for both the Mn‐PL (orange light) and Yb‐PL (NIR light) due to the light trapping events caused by the total internal reflection of the device (Figure 4D,E).^[^ ^48^, ^69^, ^71^, ^72^, ^73^, ^74^, ^75^ ^]^ In addition, the fabricated LSC device showed a high softness (Figure 4F) due to the soft feature of NC‐PDMS polymer composite,^[^ ^76^, ^77^ ^]^ demonstrating its potential to be integrated as highly flexible smart windows.^[^ ^53^ ^]^ Also, compared to previous reported PMMA polymer matrix for LSC, the PDMS matrix shows reduced C–H overtone absorption with minimal photon loss.^[^ ^46^, ^76^, ^77^ ^]^ To compare the device performance with the LSC embedded with solely Mn^2+^ doped CsPbCl_3_ NCs (denoted as Mn‐LSC), we carried out the Monte Carlo ray‐tracing simulation for both Mn‐LSC and Mn/Yb‐LSC devices with the same geometry (Figure 4G,H). While only 2.2% of the incident photons were trapped and wave‐guided to the edge region of the Mn‐LSC under the sunlight, the Mn/Yb‐LSC showed a nearly 4‐fold enhancement of the light trapping efficiency (8.7%, Figure 4G,H). This increased light trapping efficiency can be well transformed to the better device performance. Both the experimental and computer simulation results show that the internal optical efficiency (*η*_int_, Figure 4I) and external optical efficiency (*η*_ext_, Figure 4J) of the Mn/Yb‐LSC increased dramatically compared to the Mn‐LSC under sunlight illumination at different geometric gain factors (G‐factors).^[^ ^49^, ^69^, ^78^, ^79^, ^80^, ^81^ ^]^ For example, at G‐factor of 3.0, the *η*_ext_ (*η*_int_) increased from 3.1% (24.8%) for the Mn‐LSC to 9.6% (76.2%) for the Mn/Yb‐LSC under sunlight, implying a 2.1‐fold (3.1‐fold) optical efficiency enhancement. When increasing the G‐factor to 13.0, even larger enhancements of the *η*_ext_ (4.2‐fold) and *η*_int_ (5.2‐fold) were obtained (Figure 4I,J). To further characterize the LSC performances, we have integrated the Mn/Yb‐LSC and Mn‐LSC (both with a G‐factor of 12.6) to a silica photovoltaic (Si‐PV) and measured the current voltage curves (*J–V* curve) and spectrally resolved external quantum efficiencies (EQE) of the integrated devices. Both the experimental and simulation results showed a device performance enhancement of using the Mn/Yb‐LSC as compared to that of using the Mn‐LSC (Figures S16 and S17, Supporting Information), consistent with the enhanced optical efficiency (Figure 4I,J).^[^ ^80^, ^81^ ^]^

In conclusion, we describe a new category of Mn^2+^/Yb^3+^ codoped CsPbCl_3_ perovskite NCs with distinct triple‐wavelength emission at UV/blue, visible, and NIR regions. By altering the stoichiometry of the Mn^2+^‐, Yb^3+^‐, and Pb^2+^‐precursors, the emission profile can be tuned and optimized to reach a highest total PL QY of 125.3%. Our results on photoexcitation and relaxation mechanism studies show the relaxation pathways involve the energy transfer process from the host CsPbCl_3_ NCs to both Mn‐ and Yb‐dopants, as well as possible inter‐dopant energy transfer from Mn^2+^ to Yb^3+^ ion centers. We demonstrate that these Mn^2+^/Yb^3+^ codoped perovskite NCs can be used as efficient emitters for LSC applications taking advantage of their unique triple‐wavelength emission with a high PL QY and minimal reabsorption loss. Our findings make Mn^2+^/Yb^3+^ codoped CsPbCl_3_ perovskite NCs promising materials for doping chemistry studies and for various future applications.

## Conflict of Interest

The authors declare no conflict of interest.

## Supporting information

Supporting Information
