# Supplementary material for: Mn2+/Yb3+ Codoped CsPbCl3 Perovskite Nanocrystals with Triple‐Wavelength Emission for Luminescent Solar Concentrators
Source: Adv Sci (Weinh). 2020 Jul 27;7(18):2001317. doi: 10.1002/advs.202001317 (PMC7509694; doi:10.1002/advs.202001317)
Supplement: Supplementary file 1 — Supporting Information [file ADVS-7-2001317-s001.pdf]

Copyright WILEY-VCH Verlag GmbH & Co. KGaA, 69469 Weinheim, Germany, 2020.

ADVANCED SCIENCE
Open Access

Supporting Information

for *Adv. Sci.*, DOI: 10.1002/advs.202001317

Mn^{2+}/Yb^{3+} Codoped CsPbCl3 Perovskite Nanocrystals with Triple-Wavelength Emission for Luminescent Solar Concentrators

*Tong Cai, Junyu Wang, Wenhao Li, Katie Hills-Kimball, Hanjun Yang, Yasutaka Nagaoka, Yucheng Yuan, Rashid Zia, Ou Chen**

Copyright WILEY-VCH Verlag GmbH & Co. KGaA, 69469 Weinheim, Germany, 2020.

Supporting Information

Mn^{2+}/Yb^{3+} Codoped $CsPbCl_3$ Perovskite Nanocrystals with Triple-Wavelength Emission for Luminescent Solar Concentrators

*Tong Cai, Junyu Wang, Wenhao Li, Katie Hills-Kimball, Hanjun Yang, Yasutaka Nagaoka, Yucheng Yuan, Rashid Zia, Ou Chen**

Chemicals

Cesium acetate (CsOAc, 99.9%), lead (II) acetate trihydrate ($Pb(OAc)_2•3H_2O$, 99.999%), ytterbium (III) acetate tetrahydrate ($Yb(OAc)_3•4H_2O$, 99.999%), oleic acid (OA, technical grade, 90%), oleylamine (OAm, technical grade, 70%), 1-octadecene (ODE, technical grade, 90%), chlorotrimethylsilane (TMS-Cl) and nitric acid (HNO_3, 70%, trace metal basis) were purchased from Sigma Aldrich. Manganese (II) acetate tetrahydrate ($Mn(OAc)_2•4H_2O$, 99.999%) was purchased from Acros Organics. Polydimethylsiloxane (PDMS) Sylgard 184 was purchased from Dow Inc. Toluene, hexanes and ethyl acetate were purchased from Fisher. All chemicals were used as received without further purification.

Synthesis of Mn^{2+}/Yb^{3+} codoped $CsPbCl_3$ perovskite nanocrystals (NCs)

Mn^{2+}/Yb^{3+} codoped $CsPbCl_3$ NCs were prepared following a hot-injection method modified by previous report.[1] Briefly, CsOAc (53.7 mg, 0.28 mmol), $Pb(OAc)_2•3H_2O$ (75.9 mg, 0.20 mmol), $Mn(OAc)_2•4H_2O$ (4.9 mg, 0.02 mmol) and $Yb(OAc)_3•4H_2O$ (33.8 mg, 0.08 mmol) were mixed with OA (0.5 mL), OAm (1.0 mL) and ODE (5.0 mL) in a 25 mL three-neck flask. Varied Mn- and Yb- doping concentrations are achieved by changing the Mn^{2+}- and Yb^{3+}-precursor amounts during the synthesis. For samples 2-6, the feeding amount of $Mn(OAc)_2•4H_2O$ was kept unchanged (0.02 mmol), and the feeding amounts of $Yb(OAc)_3•4H_2O$ for samples 2-6 were 0, 0.02, 0.04, 0.06, 0.08 mmol, respectively. The solution was degassed for 10 min at room temperature followed by heating up to 120 °C for 1 hour. During this process, all precursors were fully dissolved, and the mixture solution became transparent. The reaction vessel was refilled with N_2 and heated to 200 °C. Once the solution reached this temperature, 0.20 mL TMS-Cl was swiftly injected, and the solution became turbid immediately. After 10 s, the solution was cooled down to room temperature by an ice bath.

Purification of Mn^{2+}/Yb^{3+} codoped $CsPbCl_3$ perovskite NCs

Firstly, the crude solution was centrifuged for 10 min at 7000 rpm. The supernatant was discarded, and the white precipitate was dissolved in 10 mL toluene. Secondly, the solutions were centrifuged again for 10 min at 4500 rpm and the clear supernatant solution was kept. The mixture was then filtered through a 0.25 μm filter. The clear solution was kept for further characterizations and measurements.

Fabrication of Mn^{2+}/Yb^{3+} codoped NC-based luminescent solar concentrators (Mn/Yb-LSCs)

The purified Mn^{2+}/Yb^{3+} codoped NC solution was dried first and then redissolved in 1 mL hexane solution and added into a mixture with 100 g of PDMS Sylgard 184 and 14 g of curing agent to achieve 0.3 wt. % of the codoped NCs with respect to the PDMS polymer matrix. The

mixture was stirred and kept under vacuum for 1h to form a homogeneous clear solution without bubbles inside. Then the mixture solution was transferred into curing molds with different dimensions. The sample was cured at 75°C for 12h in an oven. The final Mn^{2+}/Yb^{3+} codoped NCs LSCs were removed from the mold after curing.

General characterization

NC samples were dispersed in toluene for the absorption, PL, PLE, and pseudocolor map of excitation-dependent PL spectra measurements and in hexane for PL QY measurements. UV-Vis absorption spectra were measured using an Agilent Technologies Cary 5000 UV-Vis-NIR Spectrophotometer. Photoluminescence (PL) and photoluminescence excitation (PLE) spectra were measured by an Edinburgh Instruments Fluorescence Spectrometer FS5 utilizing a Xe lamp for excitation. Emission spectra were corrected for intensity and wavelength from factory provided correction files using calibrated and traceable lamps. The detectors used for measuring the PL include a visible range signal detector (a UV enhanced silicon photodiode) with spectral coverage from 230 nm to 870 nm and a NIR signal detector (a thermoelectric cooled InGaAs photodiode) with spectral coverage from 850 nm to 1650 nm. The full PL spectra were obtained by connecting two spectra collected by the visible signal detector and NIR signal detector with a scaling factor using $CuInS_2/ZnS$ core/shell nanocrystal (emitting at 737 nm with a peak width of ~ 145 nm) solution as a calibration sample for the responsivity of the two detectors under the same measurement conditions. The obtained signal scaling factor was further validated by the organic dye IR 806 (emitting at 830 nm with a peak width of ~ 30 nm). The bandgap (BG) and Mn emission PL QYs were measured directly using integrating sphere based on the equation shown below:

$$PL\ QY = \frac{N_{em}}{N_{abs}} = \frac{\int (I_{sample}(\lambda) - I_{reference}(\lambda))d\lambda}{\int (E_{reference}(\lambda) - E_{sample}(\lambda))d\lambda}$$

Where "*I*" is the intensity of the emitted light after correction, "*E*" is the intensity of excitation light after correction, "sample" means the measurements for the NC solution samples, and "reference" means the measurements for the reference sample (hexane only solution) in a quartz cuvette. The Yb PL QYs and overall PL QYs were calculated based on the obtained scaling factor and integrated PL peak areas. All the spectra are corrected in real time for intensity variances as a function of detector and grating efficiency based on the provided correction files.

X-band electron paramagnetic resonance (EPR) spectra were obtained by a Bruker EMX Premium-X EPR Spectrometer. Measurements were taken at room temperature with a 9.86 GHz microwave frequency, 4 G modulation amplitude and a power of 2 mW.

Transmission electron microscopy (TEM) characterization was performed on a JEOL 2100F operated at 200 kV. The NC sample dissolved in a toluene solution (~10 µL) was drop-casted onto a 300-mesh copper TEM grid and dried in ambient conditions.

X-ray diffraction (XRD) patterns were obtained on a Bruker D8 Discovery 2D X-ray Diffractometer equipped with a Vantec 500 2D area detector operating with Cu Kα (λ = 1.541 Å) radiation. The NC samples were drop-casted on the glass slides and evaporated under mild heating (~ 60 ºC).

X-ray photoelectron spectroscopy (XPS) measurements were performed on a Thermo Scientific K-Alpha instrument operating on Al Kα=1486.6 eV radiation with a spot size of ~ 200 µm. The NC samples were drop-casted on the silicon wafers and evaporated under mild heating (~ 60 ºC).

For inductively coupled plasma-atomic emission spectroscopy (ICP−AES) analysis, the NC solution was dried and then digested in nitric acid (~70 °C, 6 hours) to ensure complete dissolution. The solution was then diluted with 2% HNO_3 solution to suitable concentrations. The measurements were carried out on a Thermo Scientific iCAP 7400 DUO ICP−AES equipped with a Teledyne ASX-560 240 position autosampler.

Transmission spectra were measured using an Agilent Technologies Cary 5000 UV-Vis-NIR Spectrophotometer. The LSC devices were placed onto a film holder accessory for the transmission measurement.

Solution PL lifetime measurements

For BG-PL and Mn-PL lifetime, the measurements were conducted using an Edinburgh Instruments Fluorescence Spectrometer FS5 equipped with time correlated single photon counting method (TCSPC) and an EPLED-360 light source or a microsecond xenon flashlamp. The samples were dispersed in toluene within a quartz cuvette and excited at 360 nm. For Yb-PL lifetime measurement, 401 nm coherent laser cube is operated at pulsed mode, 300 Hz repetition rate, 100 ns pulse width (2.5 ns on/off switching time). Sample is illuminated under 2000 mW/cm^2 power density when laser is on. PL signals then pass through a bandpass filter (Semrock FF01-1001/234-25) and detected with single-photon avalanche photodiode (PicoQuant, τ-SPAD). Multichannel Averager (Stanford Research Systems, SR430) is used for recording time correlated single photon counting data. The decay curve was fitted with the tri-exponential decay expressed below:

$$I(t) = \sum_{k=1,2,3} I_k \exp\left(-t/\tau_k\right)$$

where $I(t)$ is the observed ensemble PL intensity at time t, and I_k and τ_k are the intensity and lifetime of an arbitrary excited state of k, respectively. We performed the fitting using the *FAST* software package (Edinburgh Instruments) and the PL intensity decays were found to be well-fitted by three exponential decay curves (k =1, 2, 3), with a goodness-of-fit (χR^2) in the range of 1.0 to 1.35.

External optical efficiency (η_{ext}), internal optical efficiency (η_{int}), and concentration factor (C factor) measurements for LSCs

Internal optical efficiency (η_{int}) is defined as the ratio of the LSC edge-collected photons to the total LSC absorbed photons from incident light, which can be obtained by measuring the LSC edge PL QY. External optical efficiency (η_{ext}) is the ratio of edge emitted photons to the total incident photons, which can be calculated by the following equation:

$\eta_{ext} = \eta_{int} \times \eta_{abs}$, (S1)

Where, η_{abs} is the absorption ratio of LSC device, which represents the portion of incident photons captured by the LSC device, described as:

$\eta_{abs} = (1-T)(1-R) = (1-R)(1-e^{-\alpha_1 d})$, (S2)

where T is the transmission of the overall LSC device, α_1 is the absorption coefficient of the LSC device, R is the reflection coefficient of LSC surface. The C factor can be obtained by multiplying the η_{ext} with the geometry gain factor (G, the ratio of the areas of the top/bottom

surface to edge regions). The overall parameter to describe LSC performances[2] is expressed as:

$$C\,factor = G \times \eta_{ext},\ \text{(S3)}$$

LSC photovoltaic (PV) integrated device characterizations

Polyscrystalline silicon (c-Si) AMX3d Micro Mini Solar Cells coupled with the edge regions of LSCs were fabricated for the devcie characterizatons. The black tape was applied to cover the excess area of the solar cell, the rest of LSC edge region and LSC bottom surface. Then, the current voltage measurements (*J-V* curves) were conducted for the whole system. The *J–V* characteristics of the solar cells, Mn-LSC and Mn/Yb-LSC were obtained using the 2400 SourceMeter (Keithley, USA) under the simulated 1-sun AM1.5G 100 $mA{\cdot}cm^{-2}$ intensity (Sol3A Class AAA, Oriel, Newport; USA) at ambient conditions, with a step size of 0.05 V and a delay time of 100 ms. The spectrally resolved external quantum efficiency (EQE) measurements were conducted using a quantum efficiency measurement system (IQE 200B; Oriel; USA) consisting of a xenon lamp, amonochromator, a lock-in amplifier, and a calibrated silicon photodetector, with a step size of 20 nm.

Monte Carlo ray-tracing simulation (MC simulation)

As a widely applied simulation approach for LSC system, MC simulation can track a number of photon traces in a LSC device.[3-6] Therefore, we can predict an overall trend of photon propagation and further evaluate the device performances. The working principle of the MC-mode is described in the following paragraphs and shown in the logic flow chart below.

Simulation inputs include: the total number of photons (i.e., 30,000) in the MC simulation, the dimension parameter (1.26 cm × 1.26 cm × 0.1 cm) of the LSC, the refractive index of the polymer matrix material (n = 1.4 for PDMS), the emission and absorption spectra and the PL QY of the emitters. Next, according to absorption data and Fresnel Law, we calculate how many of the incident random photons will be absorbed by the embedded NCs. For each captured photon, we assign random x and y coordinates for its initial position. We then determine if the absorbed photon will be emitted by the NC emitters (according to the emission spectrum and the PL QY of the perovskite NCs). If not, the photon is lost through the non-radiative decay channels of the NCs. Otherwise, we determine if the photon will hit the surface of LSC before re-absorbed by another NC. If not, the photon will be considered back to the previous emission determination step. Otherwise, the photon will be updated with a new set of coordination vectors then proceed to the next step. Based on the new photon location coordination, we can determine which surface the photon hits (top/bottom or edge of the LSC).

According to the location vectors of the photon that hits the surface and refractive index of the polymer matrix, we can determine whether the photon will be reflected through the total internal reflection (TIR). If not, the photon will transmit through the surface and close the photon tracing loop. Otherwise, the simulation procedure moves back to the initial emission determination step and surface hitting step until the loop is closed. After that, the next random photon will be released into the main loop to repeat the ray-tracing procedures. The main loop will be repeated until all the photons are traced. If the photon travels through the edge surfaces, we consider it as a collected photon; if the photon goes to the top/bottom surfaces, we consider it as a lost photon.

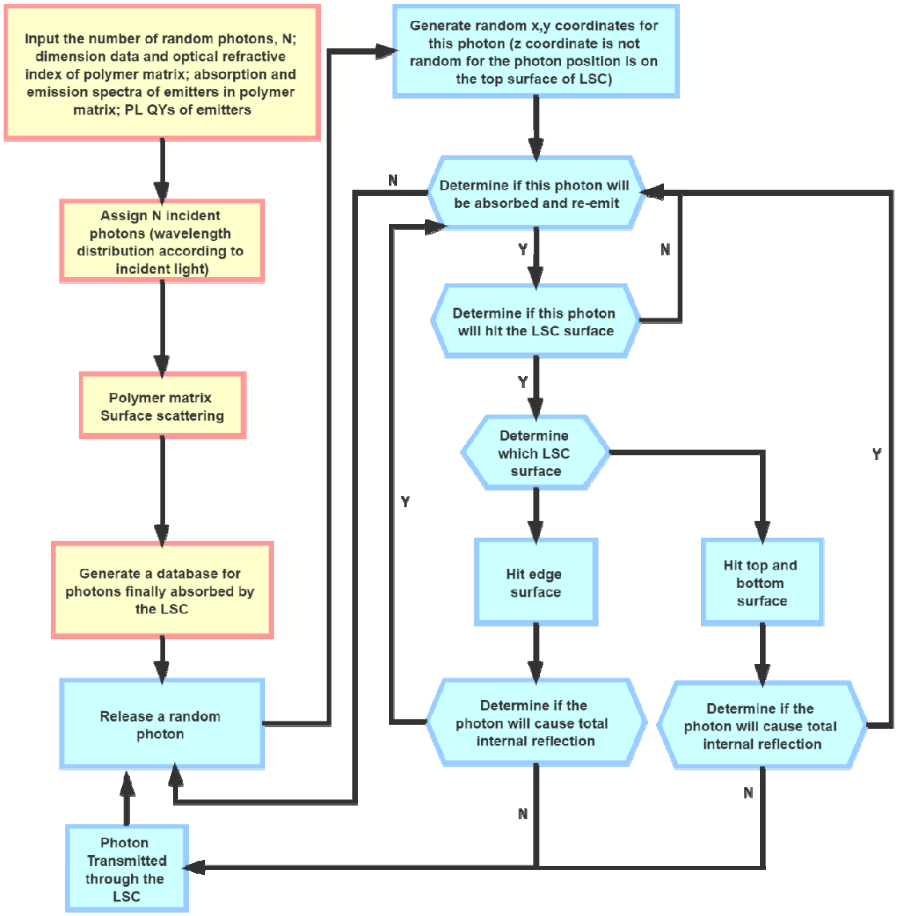

Caption: Logic flow chart of the MC simulation for LSC devices. The blue blocks are the main loop region, where individual photons are traced.

Analytical mode simulation

Based on Weber and Lambe analysis, the LSC efficiency function is expressed as below.

$$\eta_{wg}^{1} = \frac{1}{1+\beta\alpha_2 L}, \text{(S4)}$$

Here, η_{wg}^{1} is the probability of the first-time reabsorption loss, β is a geometry correction parameter (set by the equation (S12)). Then, the collection efficiency (η_{col}^{1}) of the first generation of emitted photon can be represented as:

$$\eta_{col}^{1} = \eta_{wg}^{1}\eta_{PL}\eta_{trap}, \text{(S5)}$$

η_{PL} is the PL QY of emitter, η_{trap} is the light trapping efficiency which is defined as:

$$\eta_{trap} = \sqrt{1-n^{-2}} = cos\theta_{esc}, \text{(S6)}$$

Where, n is the LSC's refractive index, θ_{esc} is the escape cone angle, which is defined by Snell's law as $\theta_{esc} = \arcsin\left(\frac{1}{n}\right)$. To account for the collection efficiency (η_{col}^{2}) of the second-generation of re-emitted photons, the function is expressed as follow:

$\eta_{col}^2 = \eta_{PL}\eta_{trap}(1-\eta_{wg}^1)\eta_{col}^1$, (S7)

Thus, each re-emission process term represents a member of a geometric progression. The total collection efficiency (η_{col}) can be expressed as a sum of contributions due to all photon generations.

$\eta_{col} = \sum_{i=1}^{\infty}\eta_{col}^{i}$, (S8)

However, the Weber and Lambe analysis do not consider any scattering effect. This can generate considerable errors for the re-absorption free systems like the LSC in this case. Therefore, by considering the scattering and re-absorption free effect, the η_{int} and η_{ext} calculations can be expressed as follows:

$\eta_{int} = \frac{\eta_{PL}\eta_{trap}}{1+\beta S_2 \mathrm{L}(1-\eta_{trap})}$, (S9)

$\eta_{ext} = \frac{(1-R)(1-e^{-\alpha_1 d})\eta_{PL}\eta_{trap}}{1+\beta S_2 \mathrm{L}(1-\eta_{trap})}$, (S10)

Where, S_2 is the scattering coefficient at the PL wavelength, L is the edge length of LSC. $<\alpha_1>$ is the average absorption coefficient among the incident light wavelength range represented as:

$< \alpha_1 >= -\frac{1}{d} ln\left(\frac{\int_{E_g}^{\infty} S_{in}(\lambda)e^{-\alpha(\lambda)d}d\lambda}{\int_{E_g}^{\infty} S_{in}(\lambda)d\lambda}\right)$, (S11)

The obtained value can be applied for the α_1 in the equation (S9). α is the absorption coefficient of the emitter. λis the wavelength. *d* is the thickness of the LCS. E_g is the bandgap of the emitter. S_{in} is the spectral shape of incident light.

For β value determination, the initial value can be calculated based on the following equation:

$\beta = 1.4 + 0.5\left(\frac{L}{150}\right)^{0.5}$, (S12)

Where *L* is the edge length for square-shaped LSCs.[7-9]

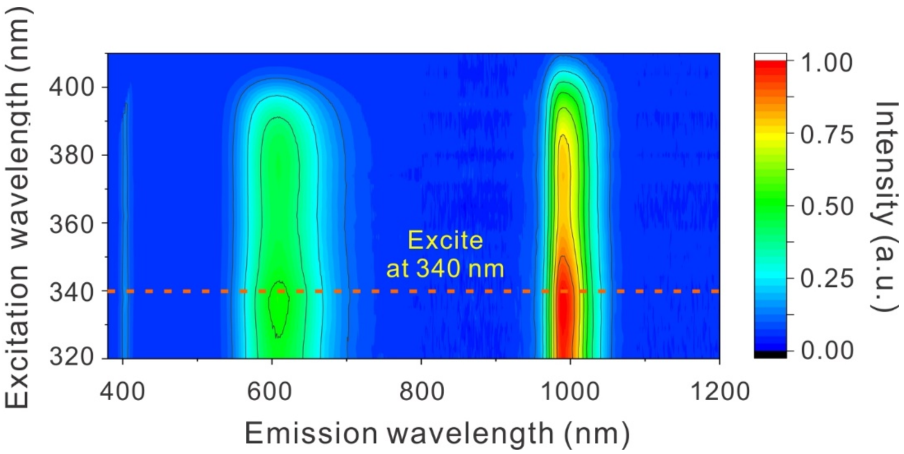

Figure S1. Pseudo-color map of excitation-dependent PL spectra of Mn^{2+}/Yb^{3+} codoped $CsPbCl_3$ perovskite NCs.

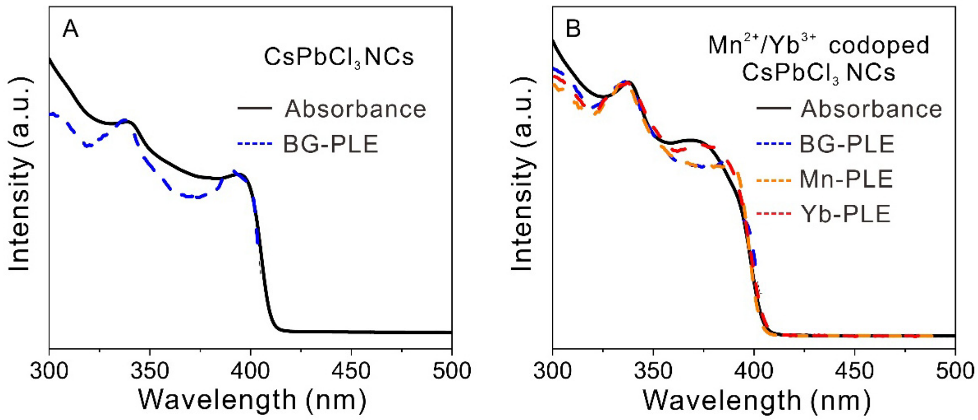

Figure S2. PLE spectra and absorption spectra of (A) undoped and (B) Mn^{2+}/Yb^{3+} codoped $CsPbCl_3$ perovskite NCs.

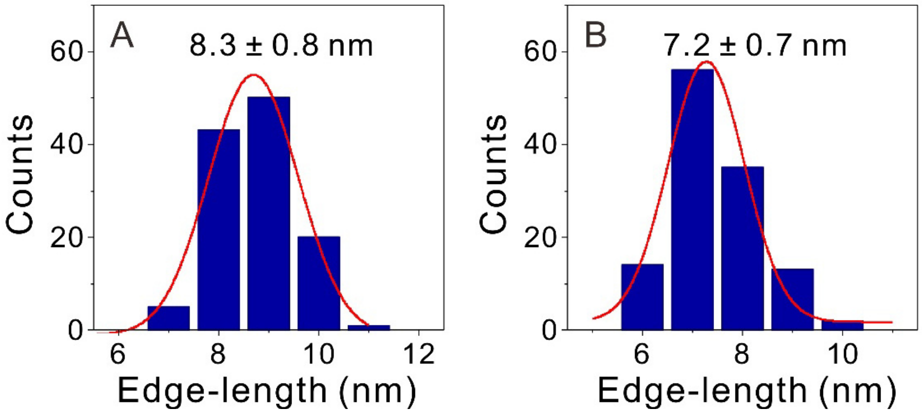

Figure S3. The size distribution histograms of (A) undoped $CsPbCl_3$ NCs (sample 1 in the main text) with an average edge-length of 8.3 ± 0.8 nm, and (B) Mn^{2+}/Yb^{3+} codoped $CsPbCl_3$ NCs (sample 5 in the main text) with an average edge-length of 7.2 ± 0.7 nm.

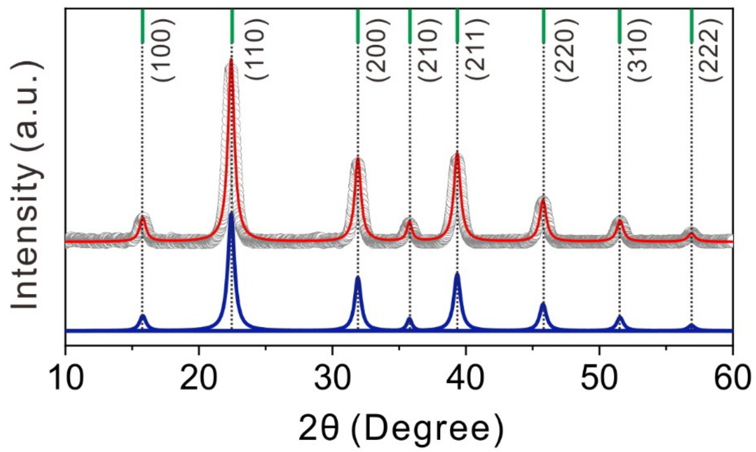

Figure S4. XRD pattern of the undoped $CsPbCl_3$ perovskite NCs (sample 1 in the main text). The original pattern, the fitted pattern, and the constituent peaks are shown in grey, red, and blue, respectively. Theoretical peak positions for $CsPbCl_3$ perovskite (space group: $Pm\bar{3}m$) are labeled with green bars. The fitting details are listed in Table S1 below.

Table S1. Fitting results for the XRD pattern of cubic $CsPbCl_3$ perovskite NCs (sample 1 in the main text).

Peak number	Assigned peak/ d(hkl)	Peak position / °	d-spacing / Å	Calculated lattice constant / Å
Peak 1	d(100)	15.79	5.611	5.611
Peak 2	d(110)	22.42	3.966	5.609
Peak 3	d(200)	31.91	2.805	5.609
Peak 4	d(210)	35.77	2.510	5.613
Peak 5	d(211)	39.35	2.290	5.609
Peak 6	d(220)	45.78	1.982	5.606
Peak 7	d(310)	51.54	1.773	5.607
Peak 8	d(222)	56.89	1.618	5.606

Average lattice constant: 5.609 ± 0.002 Å.

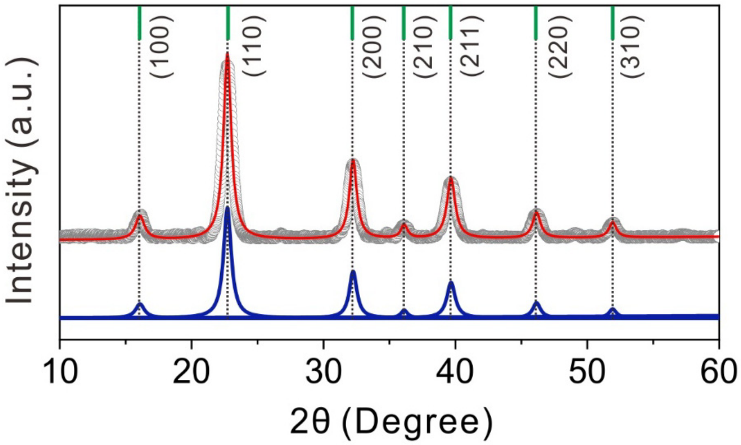

Figure S5. XRD pattern of $CsPbCl_3$: Mn^{2+} (1.30 %), Yb^{3+} (10.76 %) NCs (sample 5 in the main text). The original pattern, the fitted pattern, and the constituent peaks are shown in grey, red, and blue, respectively. Theoretical peak positions for $CsPbCl_3$ perovskite (space group: $Pm\bar{3}m$) are labeled with green bars. The fitting details are listed in Table S2 below.

Table S2. Fitting results for the XRD pattern of $CsPbCl_3$: Mn^{2+} (1.30 %), Yb^{3+} (10.76 %) NCs (sample 5 in the main text).

Peak number	Assigned peak/ d(hkl)	Peak position / °	d-spacing / Å	Calculated lattice constant / Å
Peak 1	d(100)	16.08	5.511	5.511
Peak 2	d(110)	22.71	3.915	5.537
Peak 3	d(200)	32.24	2.776	5.552
Peak 4	d(210)	36.12	2.487	5.561
Peak 5	d(211)	39.67	2.272	5.565
Peak 6	d(220)	46.15	1.967	5.563
Peak 7	d(310)	51.93	1.761	5.568

Average lattice constant: 5.551 ± 0.021 Å.

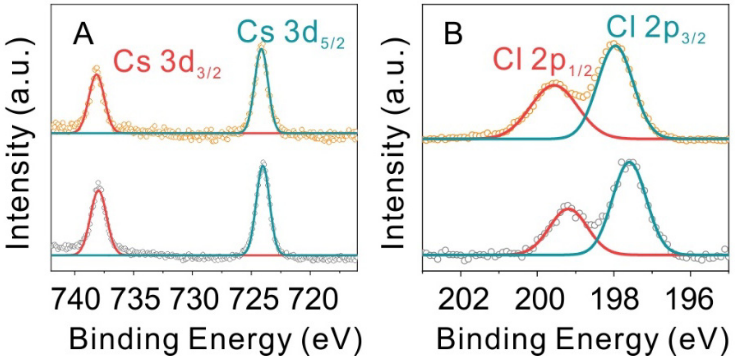

Figure S6. XPS spectra of both undoped (sample 1 in main text) and Mn^{2+}/Yb^{3+} codoped (sample 5 in main text) samples for (A) Cs 3d, (B) Cl 2p. The spectra are calibrated using C 1s peak.

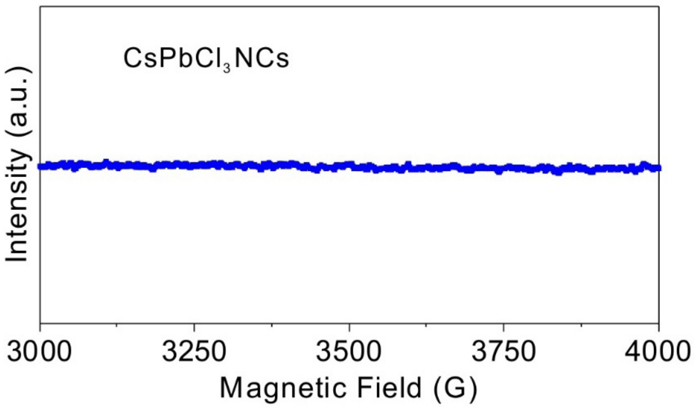

Figure S7. EPR spectrum of the undoped $CsPbCl_3$ NCs showing a complete signal silence.

Table S3. ICP-AES data analysis of the Mn^{2+}/Yb^{3+} codoped $CsPbCl_3$ NCs with different Mn^{2+} and Yb^{2+} doping concentrations.

Sample #	Pb conc. / mmol	Mn conc. / mmol	Yb conc. / mmol	Mn ratio / %	Yb ratio / %
2	6.495	0.149	0.000	2.24	0.00
3	9.368	0.217	0.393	2.17	3.94
4	5.508	0.087	0.396	1.45	6.60
5	3.610	0.053	0.442	1.30	10.76
6	5.388	0.073	0.982	1.14	15.23

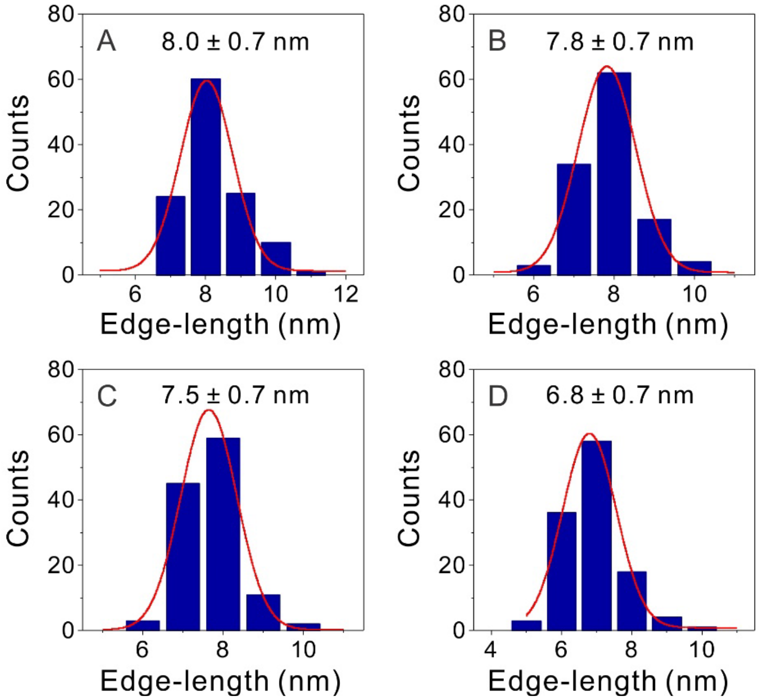

Figure S8. The size distribution histograms of Mn^{2+}/Yb^{3+} codoped $CsPbCl_3$ NCs with different doping concentrations. (A) sample 2 with an average edge-length of 8.0 ± 0.7 nm, (B) sample 3 with an average edge-length of 7.8 ± 0.7 nm, (C) sample 4 with an average-edge length of 7.5 ± 0.7 nm, (D) sample 6 with an average edge-length of 6.8 ± 0.7 nm.

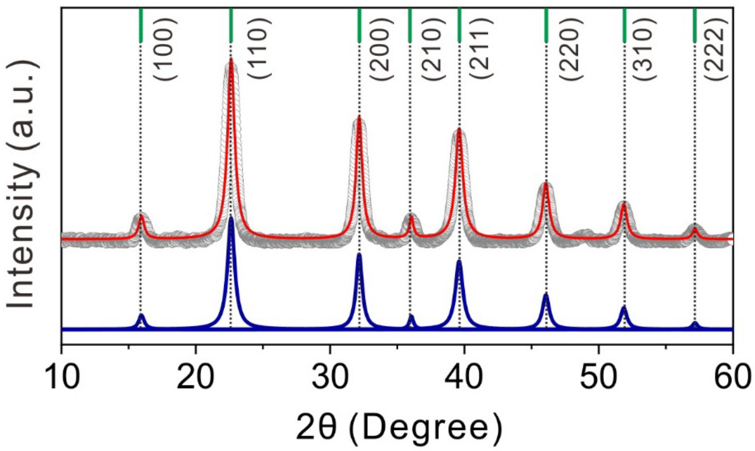

Figure S9. XRD pattern of $CsPbCl_3$: Mn^{2+} (2.24 %) NCs (sample 2 in the main text). The original spectra, the fitted spectra, and the constituent peaks are shown in grey, red, and blue, respectively. Theoretical peak positions for $CsPbCl_3$ perovskite (space group: $Pm\overline{3}m$) are labeled with green bars. The fitting details are listed in Table S4 below.

Table S4. Fitting results for the XRD pattern of $CsPbCl_3$: Mn^{2+} (2.24 %) NCs (sample 2 in the main text).

Peak number	Assigned peak/ d(hkl)	Peak position / °	d-spacing / Å	Calculated lattice constant / Å
Peak 1	d(100)	15.96	5.554	5.554
Peak 2	d(110)	22.63	3.929	5.557
Peak 3	d(200)	32.16	2.783	5.566
Peak 4	d(210)	36.05	2.491	5.570
Peak 5	d(211)	39.61	2.275	5.574
Peak 6	d(220)	46.06	1.970	5.573
Peak 7	d(310)	51.87	1.763	5.574
Peak 8	d(222)	57.17	1.611	5.582

Average lattice constant: 5.571 ± 0.008 Å.

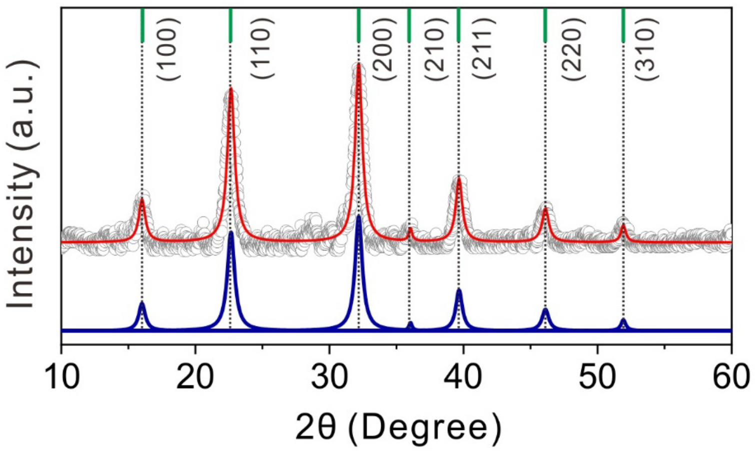

Figure S10. XRD pattern of $CsPbCl_3$: Mn^{2+} (2.17 %), Yb^{3+} (3.94 %) NCs (sample 3 in the main text). The original spectra, the fitted spectra, and the constituent peaks are shown in grey, red, and blue, respectively. Theoretical peak positions for $CsPbCl_3$ perovskite (space group: $Pm\bar{3}m$) are labeled with green bars. The fitting details are listed in Table S5 below.

Table S5. Fitting results for the XRD pattern of $CsPbCl_3$: Mn^{2+} (2.17 %), Yb^{3+} (3.94 %) NCs (sample 3 in the main text).

Peak number	Assigned peak / d(hkl)	Peak position / °	d-spacing / Å	Calculated lattice constant / Å
Peak 1	d(100)	16.02	5.534	5.534
Peak 2	d(110)	22.66	3.925	5.550
Peak 3	d(200)	32.18	2.781	5.563
Peak 4	d(210)	36.05	2.491	5.571
Peak 5	d(211)	39.67	2.272	5.565
Peak 6	d(220)	46.11	1.968	5.567
Peak 7	d(310)	51.93	1.761	5.568

Average lattice constant: 5.560 ± 0.013 Å.

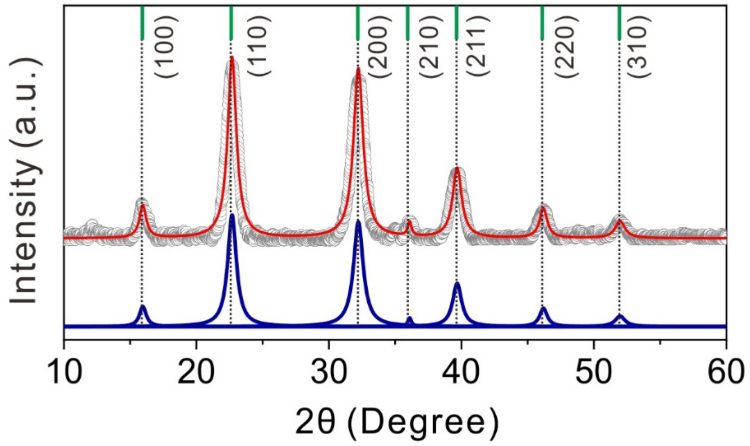

Figure S11. XRD pattern of $CsPbCl_3$: Mn^{2+} (1.45 %), Yb^{3+} (6.60 %) NCs (sample 4 in the main text). The original spectra, the fitted spectra, and the constituent peaks are shown in grey, red, and blue, respectively. Theoretical peak positions for $CsPbCl_3$ perovskite (space group: $Pm\bar{3}m$) are labeled with green bars. The fitting details are listed in Table S6 below.

Table S6. Fitting results for the XRD pattern of $CsPbCl_3$: Mn^{2+} (1.45 %), Yb^{3+} (6.60 %) NCs (sample 4 in the main text).

Peak number	Assigned peak / d(hkl)	Peak position / °	d-spacing / Å	Calculated lattice constant / Å
Peak 1	d(100)	15.96	5.554	5.554
Peak 2	d(110)	22.69	3.919	5.542
Peak 3	d(200)	32.22	2.778	5.556
Peak 4	d(210)	36.10	2.488	5.564
Peak 5	d(211)	39.68	2.271	5.564
Peak 6	d(220)	46.19	1.965	5.559
Peak 7	d(310)	51.98	1.759	5.563

Average lattice constant: 5.558 ± 0.009 Å.

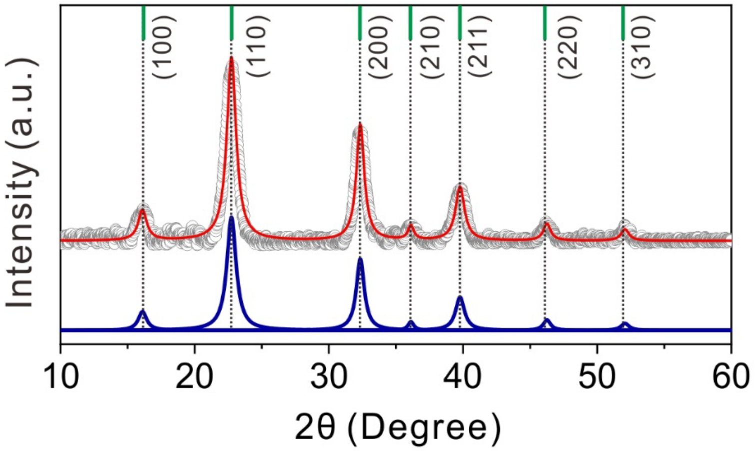

Figure S12. XRD pattern of $CsPbCl_3$: Mn^{2+} (1.14 %), Yb^{3+} (15.23%) NCs (sample 6 in the main text). The original spectra, the fitted spectra, and the constituent peaks are shown in grey, red, and blue, respectively. Theoretical peak positions for $CsPbCl_3$ perovskite (space group: $Pm\bar{3}m$) are labeled with green bars. The fitting details are listed in Table S7 below.

Table S7. Fitting results for the XRD pattern of $CsPbCl_3$: Mn^{2+} (1.14 %), Yb^{3+} (15.23%) NCs (sample 6 in the main text).

Peak number	Assigned peak / d(hkl)	Peak position / °	d-spacing / Å	Calculated lattice constant / Å
Peak 1	d(100)	16.11	5.501	5.501
Peak 2	d(110)	22.74	3.910	5.529
Peak 3	d(200)	32.35	2.768	5.535
Peak 4	d(210)	36.11	2.487	5.561
Peak 5	d(211)	39.78	2.266	5.551
Peak 6	d(220)	46.26	1.963	5.551
Peak 7	d(310)	52.07	1.756	5.554

Average lattice constant: 5.547 ± 0.012 Å.

Table S8. The summary of the PL QY for different types of Yb^{3+}-doped perovskite NCs.

Perovskite nanocrystals	PL QY (%)	Reference
$CsPbCl_3$: Yb^{3+} (5.2%)	110 (170) a)	[10]
$CsPbCl_3$: Yb^{3+} (7.4%)	130	[10]
$CsPbCl_3$: Yb^{3+} (9.1%)	142.7	[11]
$CsPbCl_{0.6}Br_{2.4}$: Yb^{3+} (1.2%)	96.4	[12]
$CsPbClBr_2$: Yb^{3+} (3.8%)	94.8	[12]
$CsPbCl_{1.5}Br_{1.5}$: Yb^{3+} (7.2%)	115.5	[12]
$CsPbCl_{1.5}Br_{1.5}$: Yb^{3+} (7.1%), Ce^{3+} (2%)	146	[12]
$CsPbCl_3$: Yb^{3+} (5.4%)	130	[13]
$CsPb(Cl_xBr_{1-x})_3$: Yb^{3+} b)	~200	[13]
$CsPbCl_3$: Yb^{3+} (6%)	164 (200) c)	[14]
$CsPbCl_3$: Yb^{3+} (10.58%)	129.1	This work
$CsPbCl_3$: Mn^{2+} (1.30%), Yb^{3+} (10.76%)	125.3	This work

a) The PL QY of 170% is measured under the very low excitation rate to avoid PL saturation effects. Under normal conditions, the PL QY was measured to be 110%.

b) The doping concentration of Yb^{3+} is not provided in the reference.

c) The PL QY of 200% is for simulation as the theoretical limit.

Table S9. Fitting results for the PL LT decay curves of the BG emission for the undoped and Mn^{2+}/Yb^{3+} codoped $CsPbCl_3$ perovskite NCs with different doping concentrations.

Sample	1	2	3	4	5	6
τ_1/ ns	0.71	0.67	0.64	0.50	0.48	0.43
percentage	35%	56%	67%	67%	73%	80%
τ_2/ ns	3.61	3.44	3.22	3.03	2.96	2.75
percentage	38%	29%	25%	25%	22%	17%
τ_3/ ns	12.45	13.09	13.79	12.76	17.42	18.20
percentage	27%	14%	8%	8%	5%	3%
$\bar{\tau}$/ ns	4.99	3.26	2.34	2.05	1.86	1.36

Table S10. Fitting results for the PL LT decay curves of the Mn emission for Mn^{2+}/Yb^{3+} codoped $CsPbCl_3$ perovskite NCs with different doping concentration.

Sample	2	3	4	5	6
τ_1/ ms	0.10	0.10	0.10	0.10	0.10
percentage	1%	2%	1%	1%	3%
τ_2/ ms	0.53	0.48	0.55	0.42	0.46
percentage	9%	14%	30%	30%	36%
τ_3/ ms	1.42	1.36	1.38	1.29	1.31
percentage	90%	84%	69%	69%	61%
$\bar{\tau}$/ ms	1.33	1.22	1.11	1.02	0.96

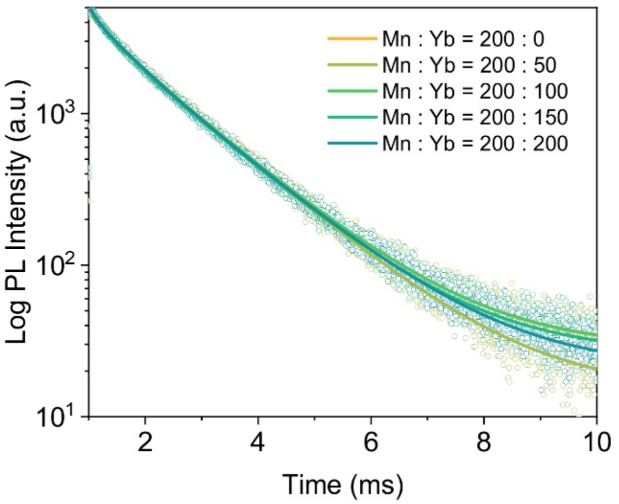

Figure S13. Lifetime decay curves of Mn-PL for the solutions mixing the solely Mn^{2+} doped $CsPbCl_3$ NCs with different amounts of solely Yb^{3+} doped $CsPbCl_3$ NCs. Mixture sample information and the fitting details are listed in Table S10 below.

Table S11. The fitting results for the PL LT decay curves of the Mn emission for the mixture of Mn^{2+} doped $CsPbCl_3$ NCs with different amounts of Yb^{3+} doped $CsPbCl_3$ NCs.

Sample	A	B	C	D	E
Mn-solution volume/ μL	200	200	200	200	200
Yb-solution volume/ μL	0	50	100	150	150
τ_1/ ms	0.10	0.10	0.10	0.10	0.10
percentage	0.8%	0.4%	0.5%	0.6%	0.9%
τ_2/ ms	0.53	0.41	0.46	0.45	0.57
percentage	9.0%	8.0%	8.9%	8.9%	10.8%
τ_3/ ms	1.42	1.40	1.41	1.29	1.42
percentage	90.2%	91.6%	90.6%	90.5%	88.3%
$\bar{\tau}$/ ms	1.33	1.32	1.32	1.31	1.32

Table S12. Fitting results for the PL LT decay curves of the Yb emission for Mn^{2+}/Yb^{3+} codoped $CsPbCl_3$ perovskite NCs with different doping concentration.

Sample	3	4	5	6
τ_1/ ms	0.29	0.02	0.04	0.12
percentage	3%	2%	4%	3%
τ_2/ ms	0.99	1.09	1.02	1.09
percentage	97%	87%	96%	97%
$\bar{\tau}$/ ms	0.97	1.11	1.14	1.42

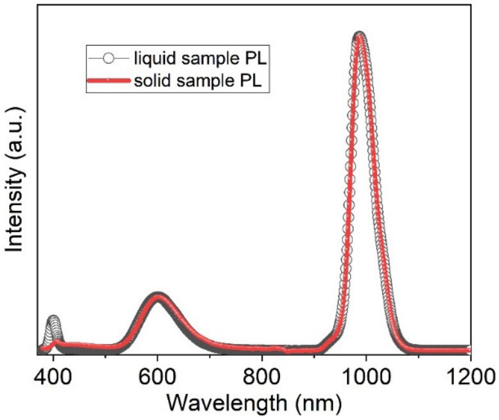

Figure S14. Comparison of PL spectra for the solution sample of Mn^{2+}/Yb^{3+} codoped $CsPbCl_3$ perovskite NCs, and the Mn/Yb-LSC device.

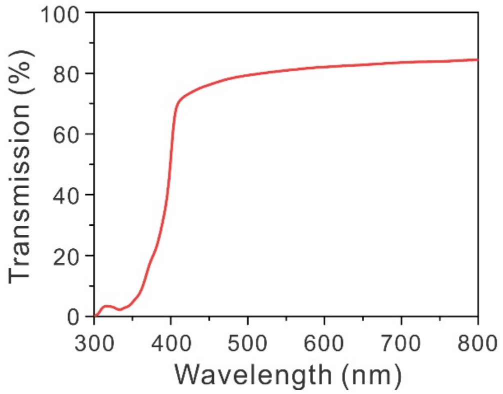

Figure S15. Transmission spectrum of the Mn/Yb-LSC fabricated using Mn^{2+}/Yb^{3+} codoped $CsPbCl_3$ perovskite NCs (LSC dimension: 3 cm * 3 cm * 0.5 cm, G = 3).

Table S13. The external optical efficiency (η_{ext}) for different perovskite NC-based LSCs at different G factors.

LSC materials	η_{ext} (%)	LSC dimensions (cm)	Reference
Mn^{2+}-doped $CsPbCl_3$ NCs	<0.5	20 ×20 ×0.5	[15]
$FAPbBr_3$ NCs	0.9	10 × 10 [a)]	[16]
Cs_4PbBr_6 NCs	2.4	10 ×10 ×0.4	[17]
Yb^{3+}-doped $CsPbCl_3$ NCs	3.7	5 ×5 ×0.2	[14]
$CsPbBr_{0.6}I_{2.4}$ NCs	2.4	10 ×10 ×0.2	[18]
$CsPbI_3$ NCs	3.1	5 × 15 [a)]	[19]
Mn^{2+}-doped $CsPbCl_3$ NCs	2.4	13 ×13 ×0.5	This work
Mn^{2+}/Yb^{3+} codoped $CsPbCl_3$ NCs	7.3	13 ×13 ×0.5	This work

a) The reference [16] and [19], only lateral dimensions of the LSC devices are provided.

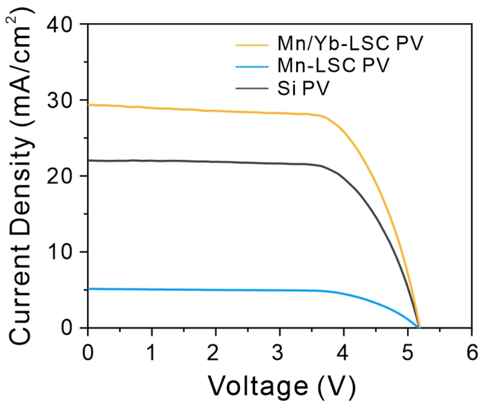

Figure S16. *J-V* responses of the Mn/Yb-LSC (Mn/Yb-LSC PV) and Mn-LSC (Mn-LSC PV) devices integrated with a Si PV, and a Si PV (Si PV) without LSC integration (LSC dimension: 12.6 cm * 12.6 cm * 0.5 cm, G = 12.6).

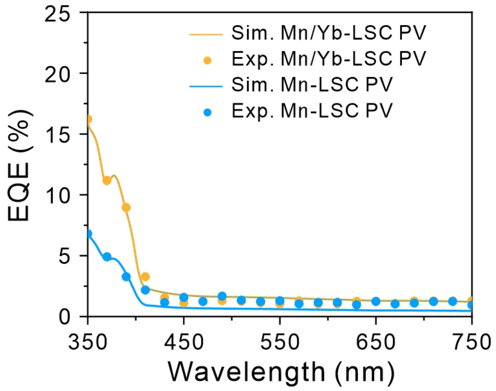

Figure S17. Experimental and simulated spectrally resolved external quantum efficiency (EQE) spectra of the Mn/Yb-LSC (yellow) and Mn-LSC (blue) coupled with Si PV (LSC dimension: 12.6 cm * 12.6 cm * 0.5 cm, G = 12.6).

References

[1] T. J. Milstein, D. M. Kroupa, D. R. Gamelin, *Nano Lett.* **2018**, *18*, 3792.
[2] H. Li, K. Wu, J. Lim, H.-J. Song, V. I. Klimov, *Nat. Energy* **2016**, *1*, 16157.
[3] I. Coropceanu, M. G. Bawendi, *Nano Lett.* **2014**, *14*, 4097.
[4] F. Meinardi, H. McDaniel, F. Carulli, A. Colombo, K. A. Velizhanin, N. S. Makarov, R. Simonutti, V. I. Klimov, S. Brovelli, *Nat. Nanotechnol.* **2015**, *10*, 878.
[5] F. Meinardi, S. Ehrenberg, L. Dhamo, F. Carulli, M. Mauri, F. Bruni, R. Simonutti, U. Kortshagen, S. Brovelli, *Nat. Photonics* **2017**, *11*, 177.
[6] F. Meinardi, A. Colombo, K. A. Velizhanin, R. Simonutti, M. Lorenzon, L. Beverina, R. Viswanatha, V. I. Klimov, S. Brovelli, *Nat. Photonics* **2014**, *8*, 392.
[7] V. I. Klimov, T. A. Baker, J. Lim, K. A. Velizhanin, H. McDaniel, *ACS Photonics* **2016**, *3*, 1138.
[8] X. Luo, T. Ding, X. Liu, Y. Liu, K. Wu, *Nano Lett.* **2019**, *19*, 338.
[9] K. Wu, H. Li, V. I. Klimov, *Nat. Photonics* **2018**, *12*, 105.
[10] T. J. Milstein, D. M. Kroupa, D. R. Gamelin, *Nano Lett.* **2018**, *18*, 3792.
[11] G. Pan, X. Bai, D. Yang, X. Chen, P. Jing, S. Qu, L. Zhang, D. Zhou, J. Zhu, W. Xu, B. Dong, H. Song, *Nano Lett.* **2017**, *17*, 8005.
[12] D. Zhou, D. Liu, G. Pan, X. Chen, D. Li, W. Xu, X. Bai, H. Song, *Adv. Mater.* **2017**, *29*, 1704149.
[13] T. A. Cohen, T. J. Milstein, D. M. Kroupa, J. D. MacKenzie, C. K. Luscombe, D. R. Gamelin, *J. Mater. Chem. A* **2019**, *7*, 9279.
[14] X. Luo, T. Ding, X. Liu, Y. Liu, K. Wu, *Nano Lett.* **2018**, *19*, 338.
[15] F. Meinardi, Q. A. Akkerman, F. Bruni, S. Park, M. Mauri, Z. Dang, L. Manna, S. Brovelli, *ACS Energy Lett.* **2017**, *2*, 2368.
[16] J. Tong, J. Luo, L. Shi, J. Wu, L. Xu, J. Song, P. Wang, H. Li, Z. Deng, *J. Mater. Chem. A* **2019**, *7*, 4872.
[17] H. Zhao, R. Sun, Z. Wang, K. Fu, X. Hu, Y. Zhang, *Adv. Funct. Mater.* **2019**, *29*, 1902262.
[18] H. Zhao, Y. Zhou, D. Benetti, D. Ma, F. Rosei, *Nano Energy* **2017**, *37*, 214.
[19] J. Wu, J. Tong, Y. Gao, A. Wang, T. Zhang, H. Tan, S. Nie, Z. Deng, *Angew. Chem. Int. Ed.* **2020**, *59,* 7738.
